# Sedation for GI Endoscopy in the Morbidly Obese: Challenges and Possible Solutions

**DOI:** 10.3390/jcm13164635

**Published:** 2024-08-08

**Authors:** Lalitha Sundararaman, Basavana Goudra

**Affiliations:** 1 Department of Anesthesiology, Brigham and Women’s Hospital, 75 Francis St., Boston, MA 02115, USA; lsundararaman@bwh.harvard.edu; 2 Department of Anesthesiology, Sidney Kimmel Medical College, 111 S 11th Street, #8280, Philadelphia, PA 19107, USA

**Keywords:** obesity, morbid obesity, nasal CPAP, high-flow nasal cannula, nasal airway

## Abstract

With the increasing prevalence of obesity and morbid obesity, this subgroup’s contribution to patients presenting for elective procedures requiring sedation is significant. Gastrointestinal (GI) procedures clearly form the largest group of such procedures. These procedures may be intended to treat obesity such as the insertion of an intragastric balloon or one or more unrelated procedures such as a screening colonoscopy and (or) diagnostic/therapeutic endoscopy. Regardless of the procedure, these patients pose significant challenges in terms of choice of sedatives, dosing, airway management, ventilation, and oxygenation. An understanding of dissimilarity in the handling of different groups of medications used by an anesthesia provider and alterations in airway anatomy is critical for providing safe sedation. Administration of sedative medications and conduct of anesthesia requires dose modifications and airway adjuncts. In this review, we discuss the above issues in detail, with a particular focus on GI endoscopy.

## 1. Introduction

Obesity has emerged as a serious, underdiagnosed, and silent pandemic, and its progress is seemingly inexorable. The World Health Organization (WHO) defines overweight as a BMI > 25 and obesity as a BMI > 30. Worldwide obesity has more than doubled since 1990, and adolescent obesity has quadrupled [1]. According to the American Heart Association, an estimated 39% to 49% of the world’s population (about 2.8–3.5 billion people) are either overweight or obese [2]. The situation is comparable in the US, with more than two in five adults considered obese (BMI of at least 30.0 kg/m^2^). More significantly, they are increasingly presenting for both surgical and non-surgical procedures. Many non-surgical procedures require sedation, frequently deep sedation bordering general anesthesia [3]. As a result, it is essential that every anesthesia/sedation provider should possess the skills necessary to manage them. Of all the sedation-requiring procedures performed annually, in the US alone, over 20 million GI endoscopic procedures are performed. Of these, approximately 6.1 million are upper endoscopies and another 15 million colonoscopies [4,5].

## 2. Pathophysiology of Obesity

### 2.1. Fat Distribution

Any excessive fat deposition is undesirable; however, visceral fat is more hazardous than peripheral subcutaneous fat deposition. It is more likely to be associated with metabolic syndrome, a constellation of central obesity, hypertension, insulin resistance, and hypercholesterolemia [6]. In addition, visceral fat deposition can lead to non-alcoholic fatty liver disease (NAFLD), and often metabolism of lipids and fat-soluble drugs may be affected in advanced NAFLD [7].

### 2.2. Respiratory Concerns

Anatomic alterations resulting from excessive fat deposition in the posterior pharynx place obese patients at greater risk of obstructive sleep apnea. It is observed that the increase in weight is directly proportional to the risk of sleep-disordered breathing [8]. Excess fat deposition can physically encroach on lung volumes and negatively affect chest movement, thus decreasing lung, chest wall, and total respiratory system compliance. Obesity is associated with increased breathing work, oxygen consumption, carbon dioxide (CO_2_) production, and increased baseline respiratory rate, thereby increasing metabolic needs. Increased basal atelectasis adds to the physiological VQ mismatch. This, coupled with decreased FRC and ERV (even in mild obesity) results in faster desaturation in the event of apnea, which frequently occurs with sedation. Co-morbidities such as sleep apnea further increase the risk of apnea, which also depends on the choice of sedative and its dosing. Indirect markers of airway closure such as RV and closing capacity are usually within normal limits in obese. However, as the FRC falls to or below the closing capacity, airway closure can occur, even with tidal ventilation [9]. The reduction in FRC is a function of the severity of obesity. Approximately 10%, 22%, and 33% reductions are seen in overweight, mildly obese, and severely obese subjects without asthma, respectively [10]. Closure of smaller alveoli and airways during spontaneous ventilation results in slower SpO_2_ recovery on return of spontaneous ventilation. Post-procedure, obese patients are at a higher risk of respiratory complications such as atelectasis, pneumonia, and respiratory failure. Proper positioning, monitoring, and lung recruitment maneuvers may be necessary to prevent such complications.

### 2.3. Cardiac Concerns

Obesity results in increased cardiac output and cardiac workload, thereby increasing blood pressure and other cardiac co-morbidity. In a meta-analysis, Bogers R.P. et al. analyzed > 300,000 adults with 18,000 Coronary Artery Disease (CAD) events. They found a positive association between overweight/obesity and the risk of developing coronary artery disease [11]. The degree and duration of obesity, expressed in BMI years, predicts better CAD than BMI alone [12]. Moreover, myocardial fat accumulation seen in obese individuals can trigger myocardial fibrosis, further leading to cardiac arrhythmias, left ventricular diastolic dysfunction, and heart failure with preserved ejection fraction (HFpEF) [13]. An HF incidence of 5% (in men) and 7% (in women) for every 1 unit of BMI is reported from the study of 5881 Framingham Heart Study participants. Such an increase was evident even after making adjustments for other risk factors and was noticeable over the entire obesity spectrum [14].

### 2.4. Thrombosis

It is well-documented that obesity is a prothrombotic state. In the Framingham offspring study (cycle 5) involving 3230 subjects (55% females, mean age 54 years) without a history of cardiovascular disease, it was demonstrated that fibrinogen, factor VII, PAI-1, and tPA antigen in both men and women (*p* < 0.001) and VWF and viscosity in women were directly related to BMI. Waist-to-hip ratio was associated with similar associations. Such association broadly persisted even after controlling for age, smoking, total and HDL cholesterol, triglycerides, glucose level, blood pressure, and use of antihypertensive medication [15]. The risk of Venous Thromboembolism (VTE) is 6.2-fold higher in obese individuals, the highest association being seen in classes II and III of obesity and patients aged >50 years [16]. The hypercoagulable may extend for up to 2 weeks after surgery, necessitating anticoagulation depending on the BMI [17].

### 2.5. Diabetes

Central obesity, as part of metabolic syndrome (syndrome X), is associated with increased insulin resistance. In addition, poor glycemic control in the peri-operative period is associated with increased morbidity, and good glycemic control is desirable [18]. The obesity paradox suggests that while patients with obesity are at greater risk of developing cardiac issues, once they do develop cardiac issues, they do better than patients with a normal BMI developing similar cardiac problems. Recent evidence indicates that the waist-to-height ratio is a better indicator of cardiac morbidity than BMI per se [19].

Zhang et al. studied the association between postoperative complications and morbidity/mortality after surgery in patients with diabetes. They discovered the existence of a hypercoagulable state with an increased risk of infections and postoperative death, but no increased risk of VTE attributable to diabetes. Nevertheless, there was no mention of obese patients with diabetes in their meta-analysis. It should be noted (as stated above) that there is an association between obesity and VTE [20].

## 3. Preoperative Concerns

### 3.1. Intravenous Access

Insertion of an IV cannula is often fraught with difficulties in obese patients due to subcutaneous fat deposition causing increased skin thickness, venous distortion, and vein visualization difficulties. In a study involving children aged 2–18 years undergoing elective noncardiac surgery, 61.8% of those classified as obese needed more than one attempt, while this was 38.7% in lean children [21]. Vein visibility and palpability were more frequent challenges in obese children than their lean counterparts. Other predictive factors of difficult cannulation are chronic illness, hypovolemia, history of IV drug abuse, vasculopathy, edema, chemotherapy, diabetes, and multiple prior hospitalizations [21,22,23,24]. Hypovolemia is common in patients presenting for a colonoscopy (because of colonic prep) and other mentioned factors frequently coexist, especially in admitted patients presenting for GI endoscopy. These factors necessitate alternative techniques and extra care during the insertion process to ensure successful access. The use of ultrasound to guide access or use of a vein visualization system is on the increase. Such visualization systems are particularly beneficial in patients with a BMI > 40, especially the female gender. By employing one such vein visualization device, 67% of subjects were successfully cannulated at the first attempt, while the success rate was 19% in the control group [25].

### 3.2. NPO Status

Despite adequate fasting, obese patients have increased residual gastric volume (>25 mL despite 8 h Nil Per Orally (NPO)) and low gastric fluid pH (<2.5) compared to non-obese individuals. Abdominal obesity increases intragastric pressure, and the risk of GERD and/or hiatal hernia formation, both of which predispose to pulmonary aspiration [26].

### 3.3. Patients on GLP 1 Agonists

Many obese patients, both diabetic and non-diabetic, are now on GLP-1 agonists. Although the evidence is sparse, there are concerns that delayed gastric emptying caused by taking GLP-1 agonists can increase the risk of regurgitation and pulmonary aspiration of gastric contents during general anesthesia and deep sedation. The American Society of Anesthesiologist guidelines recommend that patients on daily GLP 1 agonists stop taking them 1 day before, and those on a weekly schedule (injections), 1 week prior to the scheduled procedure [27]. However, a recent study from the Mayo Clinic analyzed 4134 upper endoscopies performed in 2968 unique patients taking a GLP-1 receptor agonist. They reported only two definite cases of pulmonary aspiration resulting in a cumulative incidence of 4.8 aspirations per 10,000 procedures, similar to the rate of 4.6 per 10,000 procedures in those undergoing elective upper GI endoscopy [28]. Silveira et al. noted that in 404 patients undergoing esophagogastroduodenoscopy (EGD) (33 in the semaglutide and 371 in the non-semaglutide group), semaglutide was associated with an increased residual gastric capacity but that there was a protective effect against aspiration when the EGD was combined with a colonoscopy. Moreover, they noted that there was no relationship between semaglutides and the amount of residual gastric capacity. They reported only one case of aspiration [29].

While canceling patients presenting for GI endoscopy who have not stopped GLP-1 agonists as recommended, many considerations should be contemplated. Those presenting for esophagogastroduodenoscopy (EGD) or combined EGD and colonoscopy (EGD performed first) may be safely performed. Any gastric liquid can be safely suctioned, and the procedure can proceed, while the discovery of solid/semisolid gastric contents should lead to procedure abandonment, without risking aspiration. However, other factors such as gastroparesis, achalasia, eosinophilic esophagitis, and diabetes might skew the decision towards cancellation.

### 3.4. Airway Management

Obese patients often have excess adipose tissue around the neck and upper airway, which can make emergent intubation and ventilation (in sedation cases) more challenging. Moreover, as discussed above, airway closure can occur even during resting breathing as FRC falls to levels at or below closing capacity [9]. Smaller alveoli and airways may be closed in spontaneous ventilation, and hence SpO_2_ recovery can be slow even when spontaneous ventilation resumes and patience is needed.

## 4. Drug Dosing Concerns

### 4.1. Dosing Scalars

The dosing scalars used in obese patients are displayed in Table 1. Table 2 highlights dosing considerations for commonly administered sedative/anesthesia medications in obese individuals. The following acronyms are used in these tables:

Ideal body weight (IBW), total body weight (TBW), fat-free mass (FFM), modified fat-free mass (MFFM)

### 4.2. Propofol

Obese individuals typically have disproportionately more fat mass than muscle mass. Stated differently, as their total body weight increases, the lean body mass usually remains unchanged while the fat percentage rises. Nevertheless, fat has its own blood supply, and thus, with increasing obesity, the volume of distribution shifts for many drugs. If administered based on lean body weight, induction doses of many lipophilic drugs (such as propofol) might be inadequate. While investigating awareness under anesthesia, the Association of Anaesthetists of Great Britain and Ireland and the Society for Obesity and Bariatric Anaesthesia UK found that a disproportionate number of awake patients were obese [6]. In addition, about half the awareness incidents occurred during or after induction. Similar to those with normal weight, obese patients (after a bolus of anesthetic induction agent) are unconscious at the end of one arm–brain circulation time (for propofol it is less than a minute), which depends on the cardiac output [30]. Obese individuals exhibit higher cardiac output compared to patients with lean body mass, some studies stating as high as 1 L/min, for an increase of 12.5 BMI [31]. The cardiac output is mainly distributed into non-fat tissue. The induction dose required to produce unconsciousness correlates better with lean body mass. However, lipophilic induction agents also redistribute faster and in larger amounts into the fat mass, resulting in a faster offset of induction effects. As a result, unless the infusion of propofol commences immediately and at an appropriate dose (to replenish the propofol lost to fatty tissues), there is the risk of awareness. Patients undergoing GI endoscopy can get lighter (start waking up) soon after the induction dose, leading to coughing and desaturation. Induction doses of propofol are based on lean body mass.

Propofol clearance, however, is mainly hepatic and pulmonary. It is also independent of body habitus and correlates well with TBW. As a result, the infusion of propofol should be based on TBW or modified fat-free mass. Nevertheless, with prolonged infusions, propofol can accumulate in fat, causing delayed emergence [6]. Hence the recommendation is to use LBM for induction, followed by quick initiation of maintenance dosing based on TBW, while keeping an eye on the cumulative dosing.

### 4.3. Muscle Relaxants

Nondepolarizing neuromuscular blockers are, on the other hand, distributed primarily in the central compartment, and lean body weight is an optimal dosing scalar. If TBW is used, onset time is not significantly decreased but the duration of action is prolonged. Due to the increased volume of distribution and plasma cholinesterase activity in obese patients, total body weight is appropriate for succinylcholine [32]. Doses of neostigmine and sugammadex are related to the timing and total dose of neuromuscular blocking drugs to be reversed and can usually be titrated to effect.

### 4.4. Opioids

For opioids, the clinical effect is poorly related to the plasma concentration. Dosing using lean body weight is, therefore, a sensible starting point until the patient is awake, when titration to effect is recommended. Remifentanil is often used in sedation. Due to its unique metabolism by plasma esterases, the pharmacokinetic profile of remifentanil is not substantially altered by body habitus. The volume of distribution and clearance are similar in lean and obese patients. Nevertheless, when TBW is used for dosing, excessive effect site concentrations have been noted. As a result, modified fat-free mass dosing is recommended.

## 5. Obesity and Sedation for Gastrointestinal Endoscopy

### 5.1. General Principles

Gastrointestinal (GI) endoscopic procedures are generally considered to pose minimal risk to patients. Although the practice is institution-dependent, patients above a certain BMI are usually excluded from surgicenters. The scheduling team generally contacts the pre-admission testing (PAT) clinic, and the PAT nurse approaches the anesthesiology team if deemed necessary. At this stage, any additional investigations, chiefly dictated by the associated significant comorbidities, are scheduled with a view to optimizing the procedure for the patient. Often, a nurse calls the patient and reviews the record 7 days prior to the procedure, providing necessary instructions such as colonic preparation. In some centers, the time gap between scheduling and the actual procedure day could be 18 months. On the procedure day, an updated history is obtained, the medications are verified (by both nurses and anesthesia providers), and the last doses are confirmed. This is crucial, as many endoscopic procedures require that there be no recent intake of any large pills. Some patients may benefit from preoperative antiemetics such as ondansetron or scopolamine. A physical examination is performed in the preprocedural holding area, followed by an explanation of the procedure and relevant adverse effects. A signed consent is obtained.

Subsequently, the patient is transported to the procedure room by an anesthesia provider, and all ASA-mandated monitors are attached. Most of the endoscopy procedures are conducted under monitored anesthesia care (deep sedation) unless perioperative aspiration or significant airway challenges are anticipated. The debate regarding the need for intubation during endoscopic retrograde cholangiopancreatography (ERCP) is not settled [33,34]. Although one can make a case for intubating all obese patients presenting for ERCP, the practice in the USA is very hospital-dependent [35,36,37]. At the Hospital of the University of Pennsylvania, USA, the authors demonstrated the safety of non-intubated deep sedation in morbidly obese patients undergoing GI endoscopic procedures [37]. With appropriate modification of airway adjuncts such as the nasal airway, high-flow nasal cannula, and nasal continuous positive airway devices, it is possible to perform these procedures including ERCP safely without recourse to endotracheal intubation.

### 5.2. Procedure Concerns

Obese patients may present for any of the routine and advanced endoscopic procedures or procedures specially designed to treat obesity by facilitating weight reduction. Weight loss procedures performed in obese patients could be primary (including endoscopic sleeve gastroplasty, primary obesity surgery endoluminal, insertion of intragastric balloons, aspiration therapy with the AspireAssist device, and gastric botulinum toxin injection) or revisional procedures (such as endoluminal plication in patients who underwent a Roux-en-Y gastric bypass called transoral outlet reduction). Many of these procedures are performed in the endoscopy center with an endotracheal tube (ETT), although some could be performed with deep sedation. In case of an extremely difficult airway or very severe sleep apnea, the decision may be made to secure the airway definitively with an endotracheal tube ETT at the outset itself. Extremely stimulating procedures such as double-balloon dilatation of the esophagus or dilation of a severe stricture may also warrant general anesthesia with an ETT.

In most other cases, the patient is allowed to breathe unassisted with an oxygen delivery device such as a nasal cannula, face mask, or procedural oxygen mask. Additional devices such as a nasal airway or a high-flow nasal cannula (HFNC) may be used to help with airway patency and oxygenation. In a large multicenter study involving 1994 patients, Lin et al. compared the incidence of hypoxemia and hypercarbia between patients administered oxygen with a standard nasal cannula (SNC) vs. a high-flow nasal cannula. While the incidence of hypoxemia decreased in the HFNC group, the incidence of silent hypercarbia increased. Nonetheless, a study limitation was the administration of 2 L/min of oxygen in the nasal cannula group vs. 60 L/min in the HFNC group [38]. This was somewhat corrected in the study by Mazzeffi et al., whose patient cohort included subjects with greater comorbidities including a significant smoking history, a greater percentage with Obstructive Sleep Apnea (OSA), and a higher BMI. They administered 6 L/min of SNC and 60 L/min of HFNC oxygen in patients undergoing more advanced endoscopy procedures for greater than 15 min. They measured transcutaneous CO_2,_ as well. They found that patients who received HFNC oxygen (N = 132) had a significantly lower incidence of hypoxemia than those who received NC oxygen (N = 130; 21.2% vs. 33.1%; hazard ratio [HR] = 0.59 [95% confidence interval (CI), 0.36–0.95]; *p* = 0.03). There was no difference in the incidence of hypercarbia or hypotension between the groups [39]. While using HFNCs in ERCP patients, Man-Jong Lee et al. demonstrated that unexpected hypoxia events were more frequently observed among patients in the standard nasal cannula group than among patients in the HFNC group (13% vs. 4%, odds ratio 3.41, 95% confidence interval 1.06–11.00, *p* = 0.031). The mean of the lowest oxygen saturation during ERCP was significantly lower in the standard nasal cannula group than in the HFNC group (95% vs. 97%, *p* = 0.002). As a result, HFNCs may be a useful tool in high BMI patients undergoing endoscopies. Additional caution could be exercised by measuring ETCO_2_ or transcutaneous CO_2_ [40].

**Figure 1:**
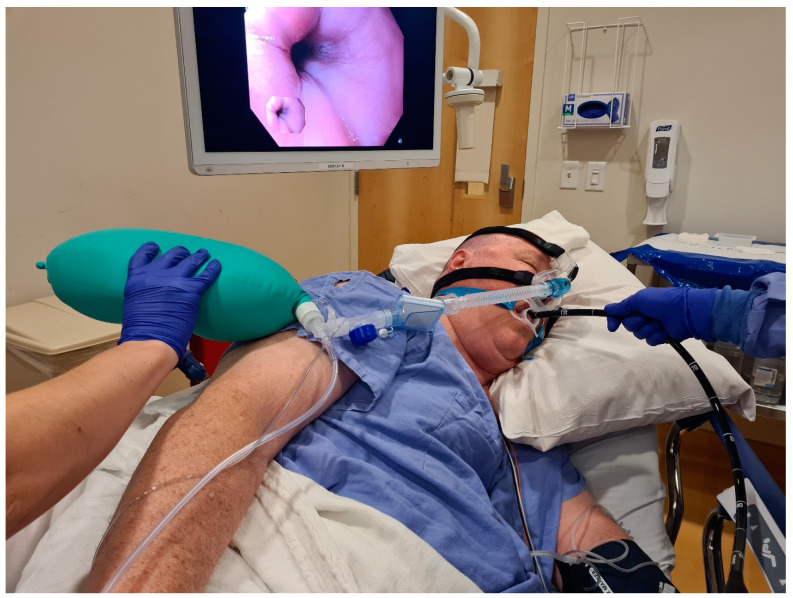
Nasal CPAP mask in use.

Nasal CPAP devices have recently been made available, and these nasal masks use positive airway pressure to stent open the upper airway (Figure 1). The airway is more likely to collapse in obese patients because of redundant fatty tissue in the posterior pharynx. Researchers have published their experiences with one such device in many situations, including EGD in obese individuals. It supposedly enables adequate preoxygenation, intra-op stabilization, and postoperative oxygenation in a single system, while allowing end-tidal CO_2_ monitoring [41,42,43]. Although the device looks promising, larger scale studies are still pending to determine their true benefit.

### 5.3. Common Medications Used in Sedation for Endoscopy

Most endoscopic procedures in developed countries are now conducted with deep sedation [44]. It has been proven that adequate sedation often results in a more relaxed procedure with greater detection of polyps and better procedural efficacy [45]. A wide variety of medications are used for sedation in upper and lower gastrointestinal endoscopy. These include but are not limited to midazolam, remimazolam, fentanyl, propofol, ketamine, remifentanil, and dexmedetomidine. While most anesthesia provider-administered sedation mainly involves propofol, conscious sedation usually involves midazolam and fentanyl. Many studies have compared patient satisfaction after anesthesiologist-administered propofol (AAP) vs. surgeon- or nurse-administered conscious sedation (NCS). Most have found greater patient satisfaction with AAP, though some have stated no significant difference [46,47]. Studies have also found greater endoscopist satisfaction with AAP vs. NCS [48]. Prolonged total room time was seen with anaesthesiologist-administered sedation with propofol vs. endoscopist-administered conscious sedation with fentanyl/midazolam [49].

With an increasing demand for AAP sedation in endoscopy, often additional adjuvants are used in sedating high BMI patients to decrease the risk of complications. In an analysis of 1172 patients undergoing advanced endoscopic procedures under sedation, Kilic et al. found that apneic episodes, desaturation episodes, and the need for airway interventions such as airway placement, suctioning, and bag-mask ventilation were more common in obese patients, especially those with a BMI >35–40 [50]. They also concluded that ketamine–propofol sedation could be used successfully by skilled anesthesia providers in this patient group.

Tuncali suggested that the addition of low-dose ketamine to midazolam–fentanyl–propofol-based sedation in patients presenting for outpatient colonoscopy results in more rapid recovery and better quality sedation. Other benefits include a reduction in propofol consumption, more stable hemodynamic status, and fewer adverse effects. The recovery times in adult patients were similar [51]. Suang yin et al. compared the efficacy of sufentanil, dexmedetomidine, and ketamine as adjuvants to propofol sedation [52]. They found that a combination of propofol + ketamine of 0.4 mg/kg maintained hemodynamic and respiratory stability, as evidenced by fewer hypotension, bradycardia, and hypoxia events in elderly patients undergoing gastrointestinal endoscopy. Zaher et al. compared a group of 40 obese patients presenting for esophagogastroduodenoscopy (BMI 30–40) who received procedural sedation with either dexmedetomidine or a ketamine-–propofol combination. They found that ketamine–propofol infusion (1:3) was a better sedation regimen for upper gastrointestinal endoscopy compared to dexmedetomidine, as less time was taken to achieve optimal sedation, with no hemodynamic instability or postprocedural complications [53].

Conscious sedation has also been studied in super-obese patients [54]. In this study, Benson et al. compared sedation for endoscopy in super-obese patients with a mean BMI of 55.2 vs. controls with a mean BMI of 22. A significantly higher percentage of obese patients experienced brief intra-procedure hypoxia (oxygen blood saturation < 90%) in comparison to the controls (5% vs. 0%, *p* = 0.02). A similar 2% of the cases and 2% of the controls experienced delayed adverse events (*p* = 1.0). Procedure completion rates were 100% for both cases and controls. They concluded that general endoscopic procedures can be safely and effectively performed in super-obese patients with moderate sedation, although brief intra-procedure hypoxia more commonly occurred in super-obese patients, and therefore higher medication doses were required. Garg et al. analyzed sedation for 7601 endoscopy procedures in 5749 obese patients. They concluded that moderate sedation was well tolerated by obese patients undergoing EGD or colonoscopy without a clinically significant difference in most procedure-related outcomes. However, their analysis included all obese (BMI > 30), without a specific focus on morbidly obese patients [55].

Wani et al. studied 1016 patients with 127 patients having a BMI > 35. They found that a higher BMI was associated with an increased need for airway maneuvers (BMI < 30, 10.5%; 30–35, 18.9%; >35–26.8%; *p* < 0.001) and hypoxemia (BMI < 30, 5.3%; 30–35, 9.4%; >35, 13.4%; *p* = 0.001) [56]. There was no difference in the frequency of need for vasopressors (*p* = 0.254) and premature termination of procedures (*p* = 0.401). In a unique study, Wang et al. investigated the effectiveness and safety of dexmedetomidine plus oxycodone for conscious sedation during colonoscopy in obese patients. They compared the combination with propofol sedation and stated that patients sedated with dexmedetomidine with oxycodone had better-preserved blood pressure, heart rate, fewer adverse effects, shorter cecal insertion time, and faster recovery times than the propofol group. The patients were also more cooperative with repositioning during colonoscopy. They, however, only included patients with a BMI of 30–35 in the study [57]. This could be an enticing sedation option in the morbidly obese, as repositioning in these patients without patient cooperation is often difficult. However, dexmedetomidine dosing would have to be carefully titrated to effect. Fentanyl and its analogs are often used as adjuvants in the sedation of morbidly obese patients. Nonetheless, care should be taken to use lean body weight for dosing, with redistribution causing variations in dose efficacy.

### 5.4. Positioning

Obese patients may pose significant challenges while being positioned on the sedation table or operating room bed, increasing the risk of pressure sores, nerve damage, and other complications. Careful planning and communication between healthcare providers are essential to ensure the patient is properly positioned and comfortable.

### 5.5. Airway Management in GI Endoscopy

While the choice of drugs and their dosing is dictated by lean body mass, ideal body weight, and total body weight, depending on the drug and timing of administration (induction or maintenance), airway management depends on the procedure requirements.

For the majority of GI endoscopic procedures, deep sedation with spontaneous ventilation is safe and effective. In a retrospective analysis of 119 morbidly obese patients undergoing a variety of GI endoscopic procedures, including many advanced procedures such as ERCP and endoscopic ultrasound, most patients’ airway management involved a nasal trumpet connected to a Mapleson breathing system [58]. One of the observations was that the frequency and severity of hypoxemia was a function of the presence or absence of obstructive sleep apnea rather than the body mass index alone [33]. In another retrospective analysis, 11,595 adult patients underwent colonoscopy or esophagogastroduodenoscopy (EGD) under deep sedation administered by either attending anesthesiologists or supervised CRNAs between June 2015 and June 2016 [59]. Their analysis showed that 314 (18%) with class I obesity, 159 (27%) with class II obesity, and 24 (19%) with class III obesity experienced hypoxemia. This contrasts with an incidence of 13% (600/11,595) in patients with normal BMI. There was no discussion of airway management.

In our experience, the following approaches are helpful in limiting the incidence and severity of hypoxemia in obese patients undergoing sedation, including for GI endoscopy.

Nasal CPAP Mask

This simple device (Figure 1) is placed on the nose and secured with a strap around the head [60,61]. It can be applied either awake or while asleep. Depending on the oxygen flow rate, it provides better oxygenation by three mechanisms. It provides very high concentrations of oxygen at the laryngeal inlet. Furthermore, it provides a degree of CPAP and consequently preserves or minimizes the reduction in FRC. Finally, by increasing the flow (either by using a flowmeter or pushing the flush oxygen valve, if connected to an anesthesia breathing system), the system allows positive pressure ventilation while the EGD is in progress. By employing this device, Jan Drews et al. demonstrated a 75% reduction in the number of patients desaturating to below 90%. Mahdi Abdallah et al. employed a nasal CPCP device in 25 obese patients with an apnea–hypopnea index (AHI) >15 undergoing functional luminal imaging probe (FLIP) assessment during EGD [62], while another 25 had standard nasal cannulas. In this retrospective study, there were 32 episodes of hypoxemia (defined as <95% sustained for a duration of more than two minutes) in the nasal cannula group, whereas in the nasal mask group, it was just one. Considering the oxygen is not humidified and to accomplish the three goals mentioned above, one needs high flows. As a result, transient dryness of the nose and throat in the post-procedure period is possible; however, any symptoms of nasopharyngeal irritation are transient.

High-Flow Nasal Cannula

High-flow nasal cannulas (HFNCs), where the oxygen flows are 50–60 L/min and administered by a special device, have been used in ICUs for many years. Their use in GI endoscopy is relatively recent. Like nasal CPAP, an HFNC provides high oxygen concentrations at the laryngeal inlet, provides CPAP, and preserves FRC. However, it does not allow positive pressure ventilation. In a meta-analysis of fifteen randomized controlled trials (seven bronchoscopies, eight gastrointestinal endoscopies), Yuan Tao et al. found that the use of HFNCs is associated with improved oxygenation, a decreased need for airway intervention, and reduced procedure interruption in patients undergoing endoscopic procedures. However, they classified the strength of evidence as moderate to low [63]. Another meta-analysis of eight RCTs concluded that the use of an HFNC did not reduce the overall incidence of hypoxemia (compared to conventional oxygen administration); however, it might reduce the incidence of hypoxemia in patients at moderate to high risk for hypoxemia [64]. Another disappointing downside of an HFNC is its ability to obscure severe respiration depression, providing an illusion of safety [65].

Nasal Airway and Mapleson Breathing System

**Figure 2:**
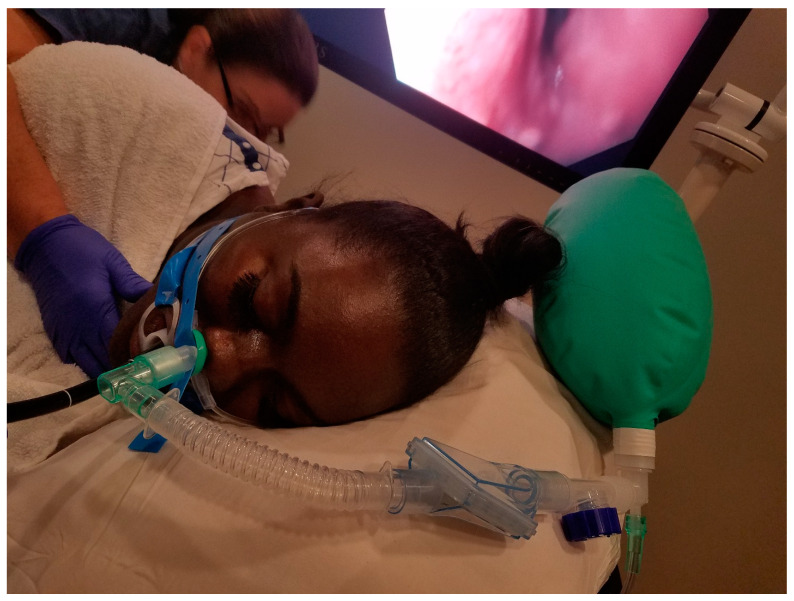
Mapleson breathing system connected to a nasal airway.

The Hospital of the University of Pennsylvania has extensive experience in the use of this technique over the last 15 years (Figure 2). One of the authors discussed their experience with this technique. Briefly, an appropriately sized soft nasal cannula (popularly called a nasal trumpet) is inserted into the nasal passage (on the more patent side, assessed before the procedure) under deep sedation and connected to a Mapleson breathing system or anesthesia breathing circuit. Oxygen flows of 6–8 L provide the same benefits as the nasal CPAP discussed above. This was found to minimize the incidence of desaturation in both routine and advanced procedures, including in the obese population [34,37].

## 6. Postoperative Concerns

The American College of Surgeons (ACS) published its National Surgical Quality Improvement Program (NSQIP) in May 2023 [66]. In an analysis of 5.6 million patients across nine surgical specialties over 7 years, the ACS National Surgical Quality Improvement Program (NSQIP) established the relationship between BMI and postoperative complications. While they did not find elevated odds of postoperative complications such as mortality, overall morbidity, pulmonary issues, and urinary tract infections among obese patients, they did find an association between obesity and higher rates of infection, VTE, and renal failure. The analysis was limited to a 30-day follow-up after surgery. Cardiorespiratory comorbidity status and metabolic syndrome sub-stratification were inadequately delineated in this analysis. Other studies came to similar conclusions [67,68]. In addition, obese patients have a 2–3 fold increased risk of coronary artery disease, and many such related events can occur up to 72 h postoperatively.

Fortunately, many GI endoscopic procedures are short and less complicated, and the patients are discharged on the same day.

## 7. Conclusions

Sedation for endoscopy in the morbidly obese patient needs a careful understanding of the altered pathophysiology, pharmacology, and anesthetic implications. Careful assessment of the airway, planning, and use of devices for airway patency and rescue are crucial. While conscious sedation and propofol sedation have been proven efficacious in the hands of an experienced sedation provider, obese patients have a greater risk of apneic episodes, desaturation, and need for airway assistance. Adjuvants such as dexmedetomidine, ketamine, fentanyl analogs, and midazolam can be used in sedation, but care must be taken to optimize the amount of anesthetic administered, keeping dosing scalars in mind.

## Figures and Tables

**Table 1:** Dosing scalars used for dosing in obese patients.

Name	Formula	Benefit	Drawback
Ideal body weight (IBW)	Male: 50 kg + 2.3 kg for each 2.54 cm (1″) over 152 cm (5 ft) Female: 45.5 kg + 2.3 kg for each 2.54 cm over 152 cm	Accounts for gender and height	Does not account for body habitus
Lean body weight (LBW)	Male: 1.1 × TBW − 128 × (TBW/Ht)2 Female: 1.07 × TBW − 148 × (TBW/Ht)2	Accounts for gender and body habitus	May underdose
Fat-free mass (FFM)	Male: (9.27 × 10^3^ × TBW)/(6.68 × 10^3^ + 216 × BMI) Female: (9.27 × 10^3^ × TBW)/(8.78 × 10^3^ + 244 × BMI)	Accounts for gender and body habitus	May underdose Fat-free
Modified fat-free mass (MFFM)	FFM + 0.5 × (TBW − FFM)	Accounts for gender and body habitus (height, LBW, and adipose weight).	Complicated

**Table 2:** Dosing considerations of commonly administered sedative/anesthesia medications in obese individuals.

Drug	Dose	Special Concerns
Propofol	Induction dose based on LBM, Infusion based on total body weight (TBW)	Induction dose based on LBM may get redistributed quickly, maintenance anesthesia must be started quickly
Suxamethonium	TBW	Plasma cholinesterase is also proportionately increased in obesity.
Non-depolarizing neuromuscular blockers	LBM	Dosing based on TBW will increase duration of action.
Remifentanil	MFFM	Though kinetic profile of drug not altered by habitus, effect site concentration increased by TBW dosing.
Fentanil, sufentanil	LBM	Clinical effect is poorly related to the plasma concentration. Dosing using lean body weight is recommended until the patient is awake and titration to effect is possible.
Remimazolam	TBW, MFFM	Though TBW is used for dosing, effect site concentration better regulated with MFFM dosing.
